# Microbiomes associated with *Coffea arabica* and *Coffea canephora* in four different floristic domains of Brazil

**DOI:** 10.1038/s41598-023-45465-w

**Published:** 2023-10-28

**Authors:** Tomás Gomes Reis Veloso, Marliane de Cássia Soares da Silva, Taís Rizzo Moreira, José Maria Rodrigues da Luz, Aldemar Polonini Moreli, Maria Catarina Megumi Kasuya, Lucas Louzada Pereira

**Affiliations:** 1 Departamento de Microbiologia, Universidade Federal de Viçosa, Laboratory of Mycorrhizal Associations – LAMIC, Avenida PH Rolfs S/N, Viçosa, CEP, Minas Gerais, 36570-000 Brazil; 2 Universidade Federal do Espírito Santo. Centro de Ciências Agrárias e Engenharias. Av. Gov. Lindemberg, 316 - Centro, Jerônimo Monteiro, CEP, Espírito Santo, 29550-000 Brazil; 3 Instituto Federal do Espírito Santo. Coffee Design. Avenida Elizabeth Minete Perim, S/N, Bairro São Rafael, Venda Nova do Imigrante, CEP, Espírito Santo, 29375-000 Brazil

**Keywords:** Microbial communities, Microbial ecology, Soil microbiology

## Abstract

Brazilian coffee production relies on the cultivation of *Coffea arabica* and *Coffea canephora*. Climate change has been responsible for the decreasing yield of the crops in the country yet the associated microbial community can mitigate these effects by improving plant growth and defense. Although some studies have tried to describe the microorganisms associated with these *Coffea* species, a study that compares the microbiome on a wider spatial scale is needed for a better understanding of the *terroir* of each coffee planting region. Therefore, our aim was to evaluate the microbial communities harbored in soils and fruits of these *Coffea* species in four Brazilian floristic domains (Amazon, Atlantic Forest Caatinga, and Cerrado). One hundred and eight samples (90 of soil and 90 of fruits) were used in the extraction and sequencing of the fungal and bacterial DNA. We detected more than 1000 and 500 bacterial and fungal genera, respectively. Some soil microbial taxa were more closely related to one coffee species than the other species. *Bacillus bataviensis* tends to occur more in arid soils from the Caatinga, while the fungus *Saitozyma* sp. was more related to soils cultivated with *C. arabica*. Thus, the species and the planting region (floristic domain) of coffee affect the microbial composition associated with this crop. This study is the first to report microbial communities associated with coffee produced in four floristic domains that include sites in eight Brazilian states. Data generated by DNA sequencing provides new insights into microbial roles and their potential for the developing more sustainable coffee management, such as the production of biofertilizers and starter culture for fermentation of coffee cherries.

## Introduction

*Coffea arabica* and *Coffea canephora* are the main species of coffee cultivated in Brazil and worldwide. *C. canephora* is cultivated at altitudes ranging from 50 to 550 m above see level (asl) and is responsible for about 25% of the total national production of coffee. *C. arabica* represents about 75% of the total production and is grown at higher altitudes (600–1200 m asl)^1^.

Many factors contribute to the success of coffee production. These include pre- and post-harvest factors. Among the pre-harvest factors, the microbiota associated with fruits and soils are key drivers to achieve high-quality production. In soils, for example, arbuscular mycorrhizal fungi (AMF) and nitrogen-fixing bacteria can improve the nutrient uptake by plants by delivering reduced nitrogen and solubilizing phosphate, otherwise unavailable to plants^2^. Appropriate crop management can improve the effect of these beneficial microorganisms on plant growth and allow coffee producers to minimize the use of chemical fertilizers and pesticides, which has been an increasing demand of the market and has environmental impacts^3^.

The strategy of revealing the microorganisms associated with soil and fruits of coffee species is a crucial step to allow the prospecting of beneficial microorganisms that can be used as biofertilizers in soils and starter cultures for improving coffee beverage quality^4,5^. The coffee cultivars are more adapted to some regions than others and their associated microorganisms can also be shaped by the climatic and edaphic traits, which is related to the concept of *terroir*^6,7^. Thus, uncovering the microbial diversity associated with the fruits and soils is the first step that provides valuable information about Plant Growth-Promoting Microbes as well as new fermenting yeasts or bacteria^8,9^.

**Figure 1:**
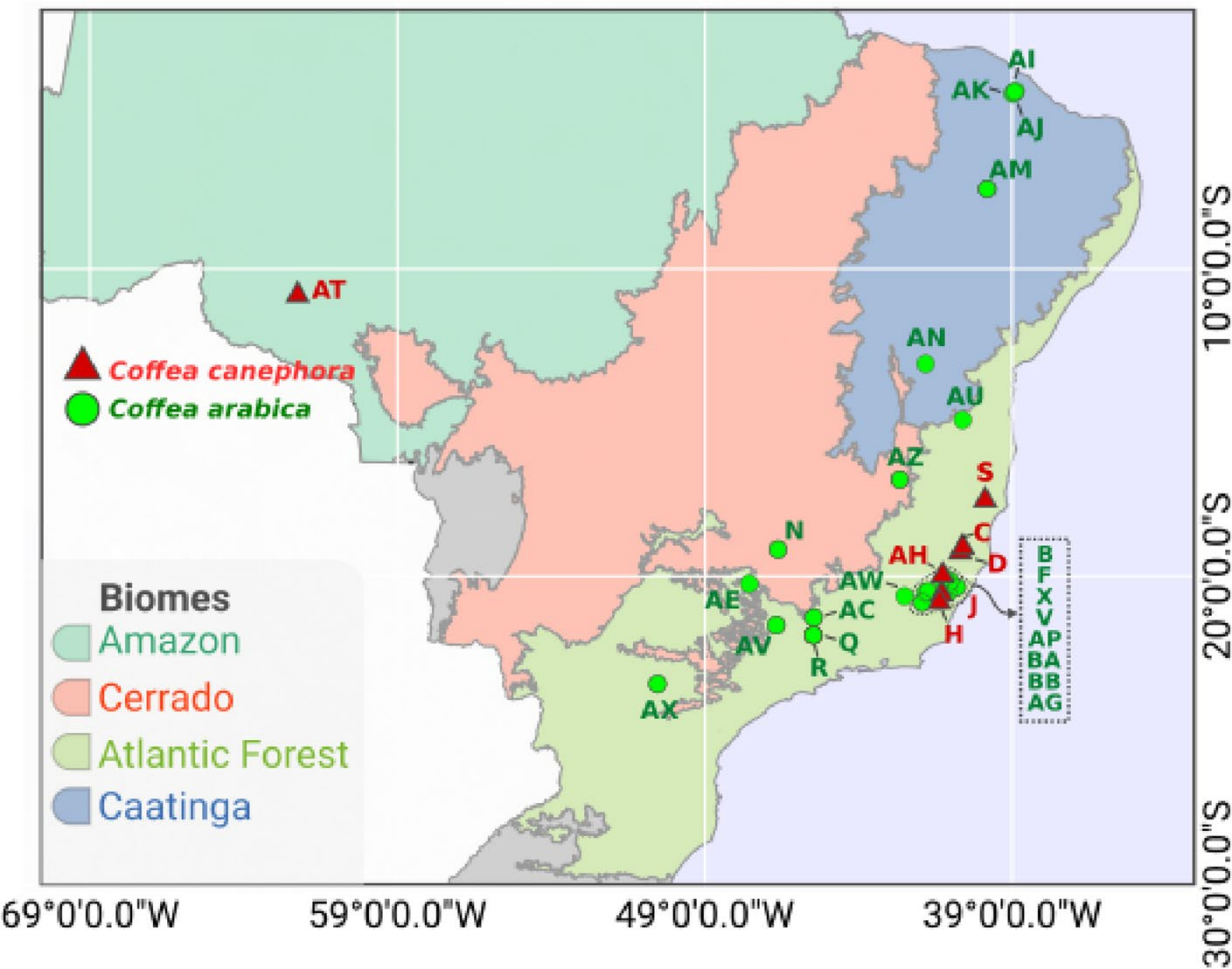
Geographic location of each sample used in this study. Ninety samples from two coffee species (*Coffea arabica* and *Coffea canephora*) were obtained from 30 properties (Table 1) across four Brazilian floristic domains (Amazon, Atlantic Forest, Caatinga, and Cerrado). Map generated with QGIS 3.26.1 'Buenos Aires’ (https://qgis.org/en/site/index.html).

Therefore, the aim of this study was to characterize, by Next Generation DNA sequencing, the microbial communities associated with soils and fruits of *C*. *arabica* and *C. canephora* produced in four Brazilian floristic domains (Amazon, Atlantic Forest, Caatinga, and Cerrado; Fig. 1). This approach allows a better understanding of the core microbial communities associated with these coffee species, the uniqueness of some coffee *terroir*, and adaptation of these plants to floristic domains with different soil and climatic conditions. In addition, the information of bacteria and fungi harbored by fruits of each coffee species can guide the prospecting of new inoculums that improve the sensory profiles of beverages of each coffee species.

## Results

### General information

**Figure 2:**
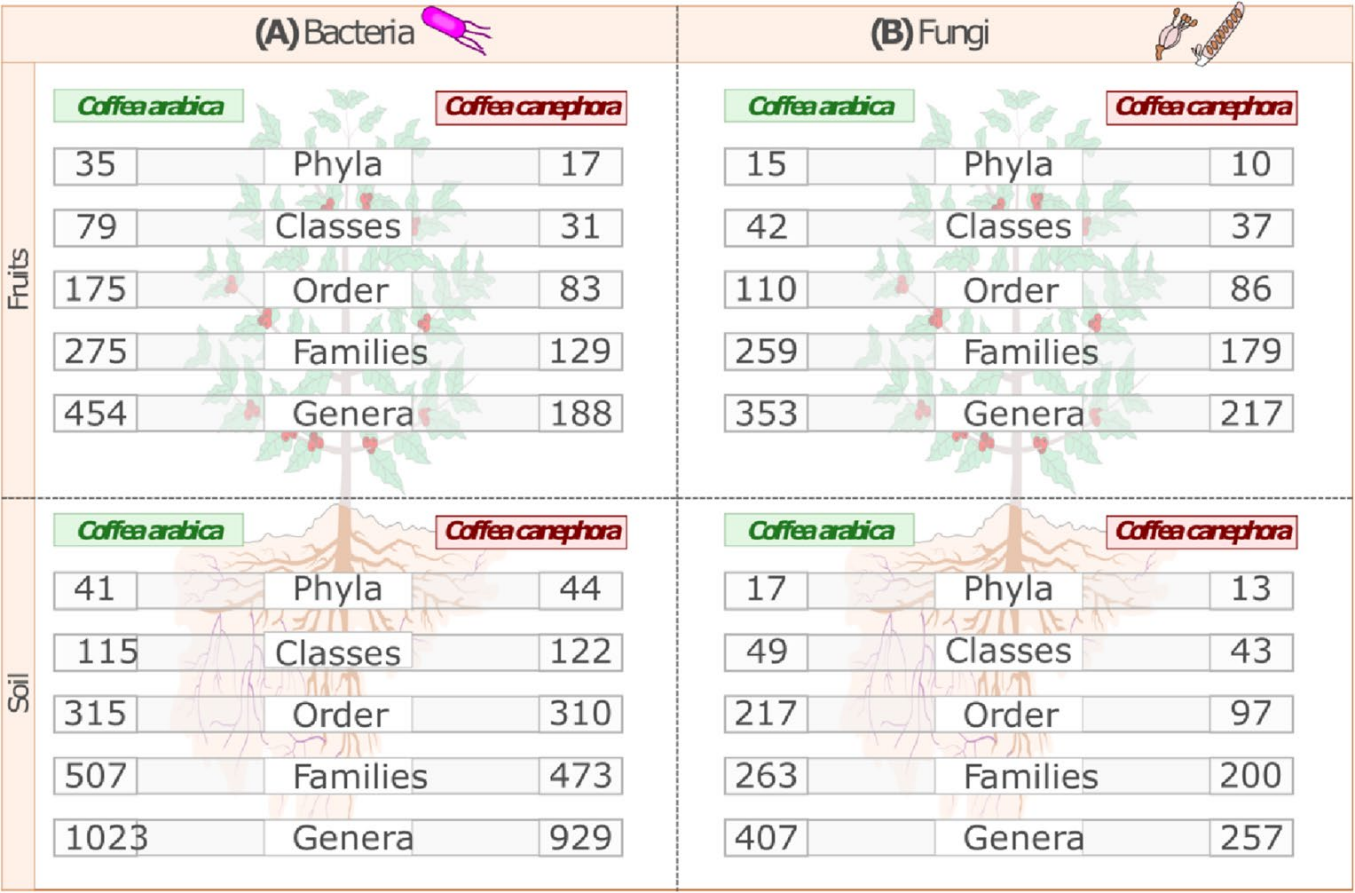
Overview of the sequencing number obtained by sequencing of the bacterial and fungal communities from soil and fruits of *C. arabica* and *C. canephora.*

In total, more than 60 million reads were obtained: 30,700,327 for 16S rDNA/bacteria and 29,920,072 for ITS/fungi. These sequences were distributed across more than 1000 bacterial and fungal genera (Fig. 2). The coverage index^10^ was above 0.95 for all samples, showing that the sequencing effort was enough to capture the microbial diversity in all samples (Supplementary Table S1). All the raw reads were deposited in the SRA archive of NCBI and are available under the BioProject PRJNA626678.

### Microbial diversity

**Figure 3:**
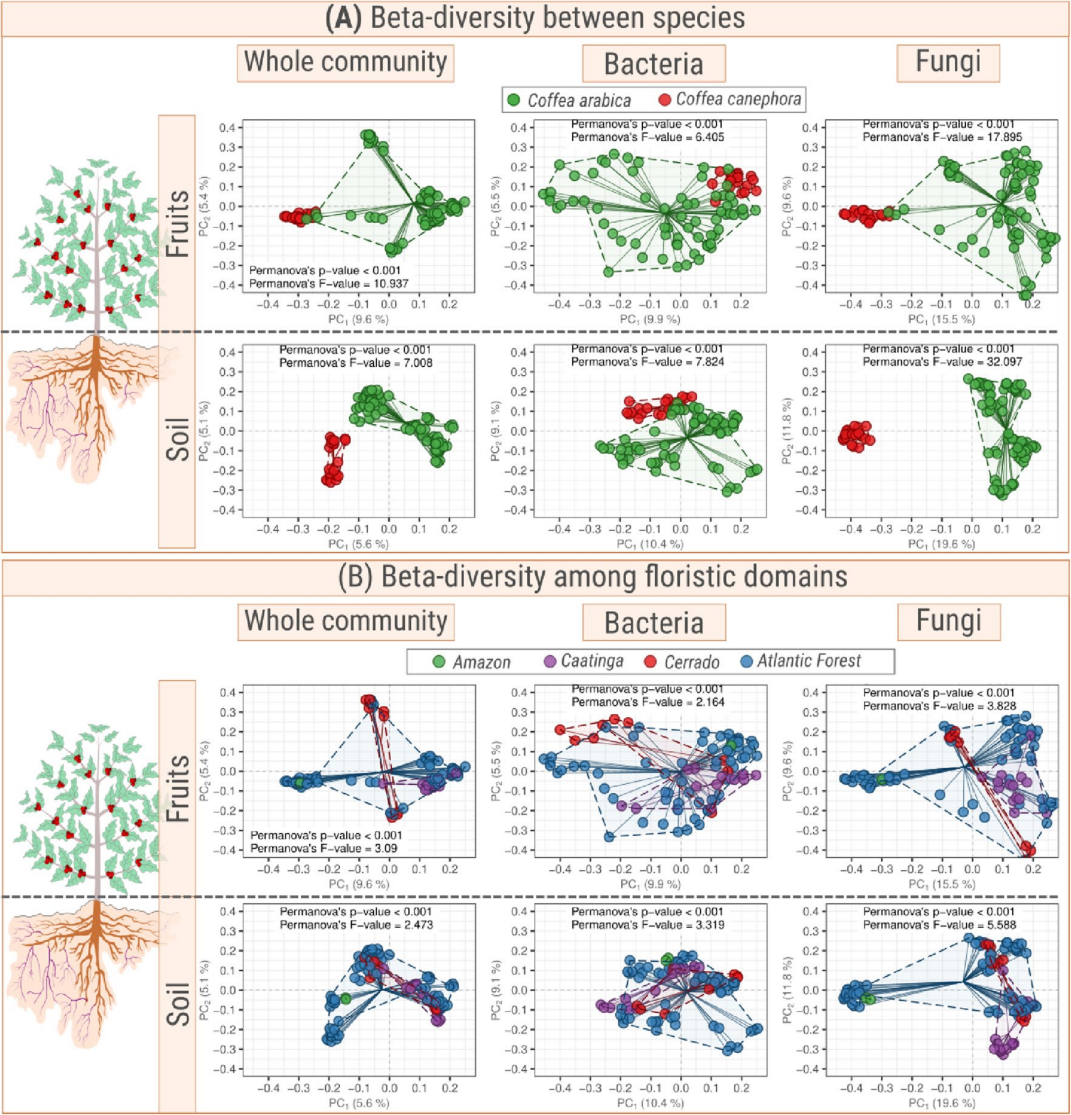
Beta-diversity of the whole community (bacteria + fungi; left plot), bacterial (middle plot) and fungal (right plot) communities harbored in fruits and soils of coffee plants from two species: *Coffea arabica* and *Coffea canephora*. The upper panel (**A**) shows the difference between microbial beta-diversities associated with the soil of each coffee species (*C. arabica* or *C. canephora*), while the lower panel (**B**) represents the differences among floristic domains. The PERMANOVA F-statistics were calculated based on 999 permutations. The higher the value of F-statistics, the greater the difference of microbial communities between (**A**) coffee species or (**B**) floristic domains.

The bacterial communities inhabiting fruits and soils of coffee plants differ between the host species (*C. arabica* vs. *C. canephora*, Fig. 3A). This difference is greater between the species than among the floristic domains (see F-statistics in Fig. 3B). By the PERMANOVA *p*-values, the fungal communities in coffee fruits were about two times (17.832/6.752 = 2.64) higher than the bacterial communities. Nonetheless, in soils the divergence was more than four times (17.8329/6.752 = 4.41) greater for the fungal community. These results show that the rhizospheric mycobiome is very different between the coffee species (Fig. 3, Fig. S1).

**Figure 4:**
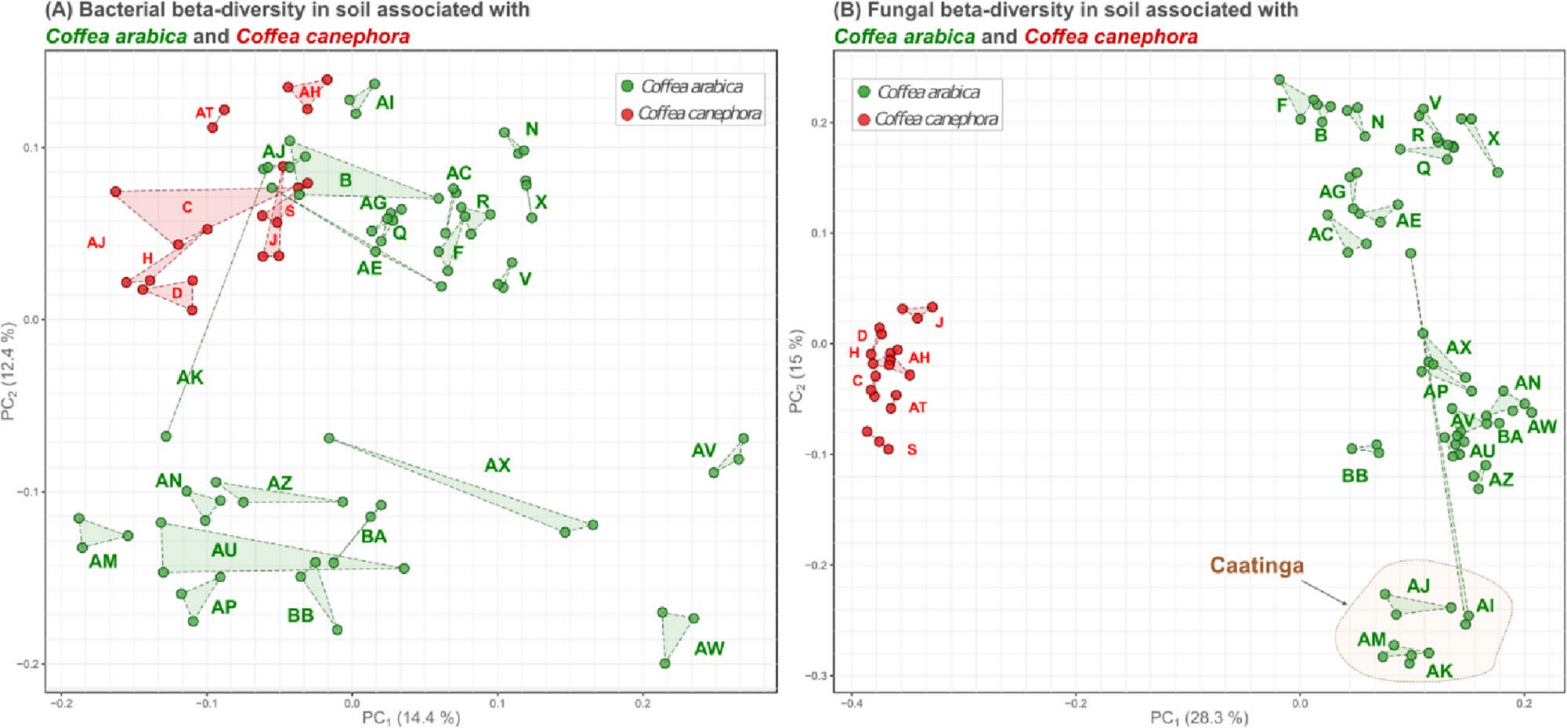
Beta-diversity of (**A**) fungi and (**B**) bacteria found in soil of *C. arabica* and *C. canephora* plantations. Each plantation is represented by three points connected by dashed lines. Each point represents the positions in the two first coordinates of the Principal Coordinate Analysis (PCoA) built from the Bray–Curtis distance matrix.

Besides the difference between the microbiomes of the two coffee species (Fig. 3B), the bacterial communities from the Atlantic forest, i.e. Araponga (site AW) and São Paulo (site AV), had a distinct community composition (Fig. 4A). In addition, the fungal communities associated with *C. arabica* of the Caatinga were distinct from those of other locations (Fig. 4B). This result may be due to *terroir* of the Caatinga floristic domains, which had low annual precipitation, high temperatures throughout the year, and shallow and stony soil.

**Figure 5:**
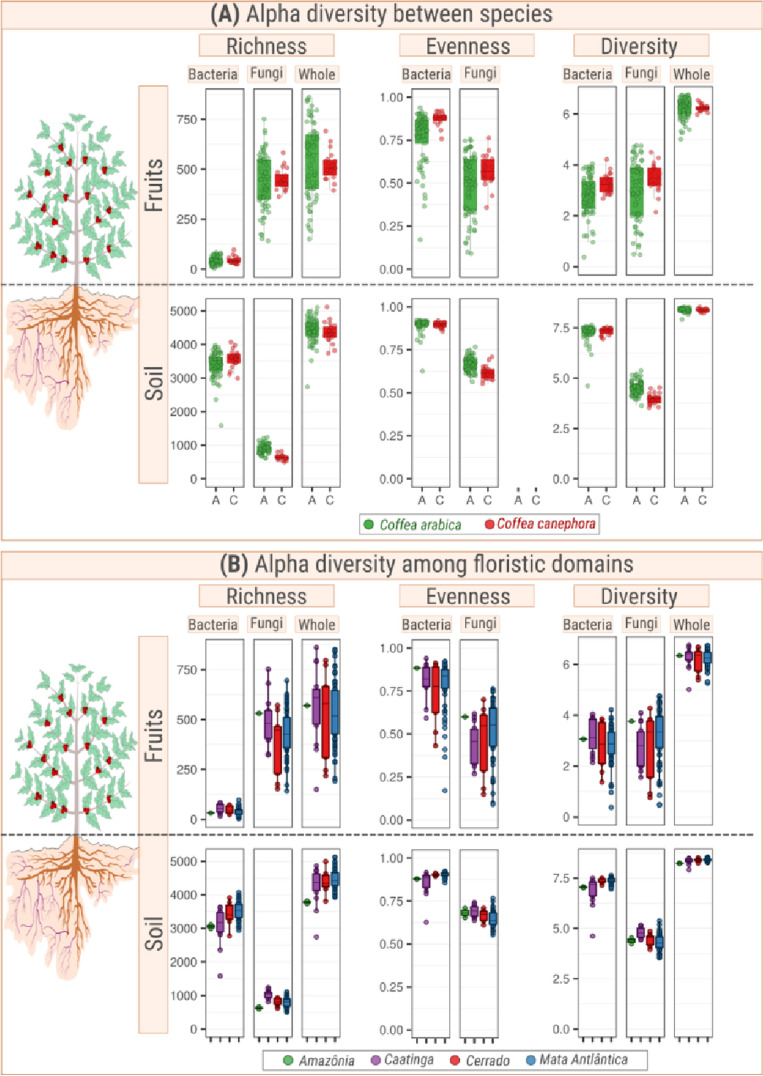
Alpha-metrics of whole community (bacteria + fungi; left plot), bacterial (middle plot) and fungal (right plot) communities harbored in fruits and soils of coffee plants from two species: *Coffea arabica* and *Coffea canephora*. The upper panel (**A**) shows the difference in microbial alpha diversity metrics between coffee species (*C. arabica* or *C. canephora*), while the lower panel (**B**) represents the differences among floristic domains. The PERMANOVA F-statistics were calculated based on 999 permutations. The higher the value of F statistics, the greater the effect of the species or floristic domains on the community.

**Figure 6:**
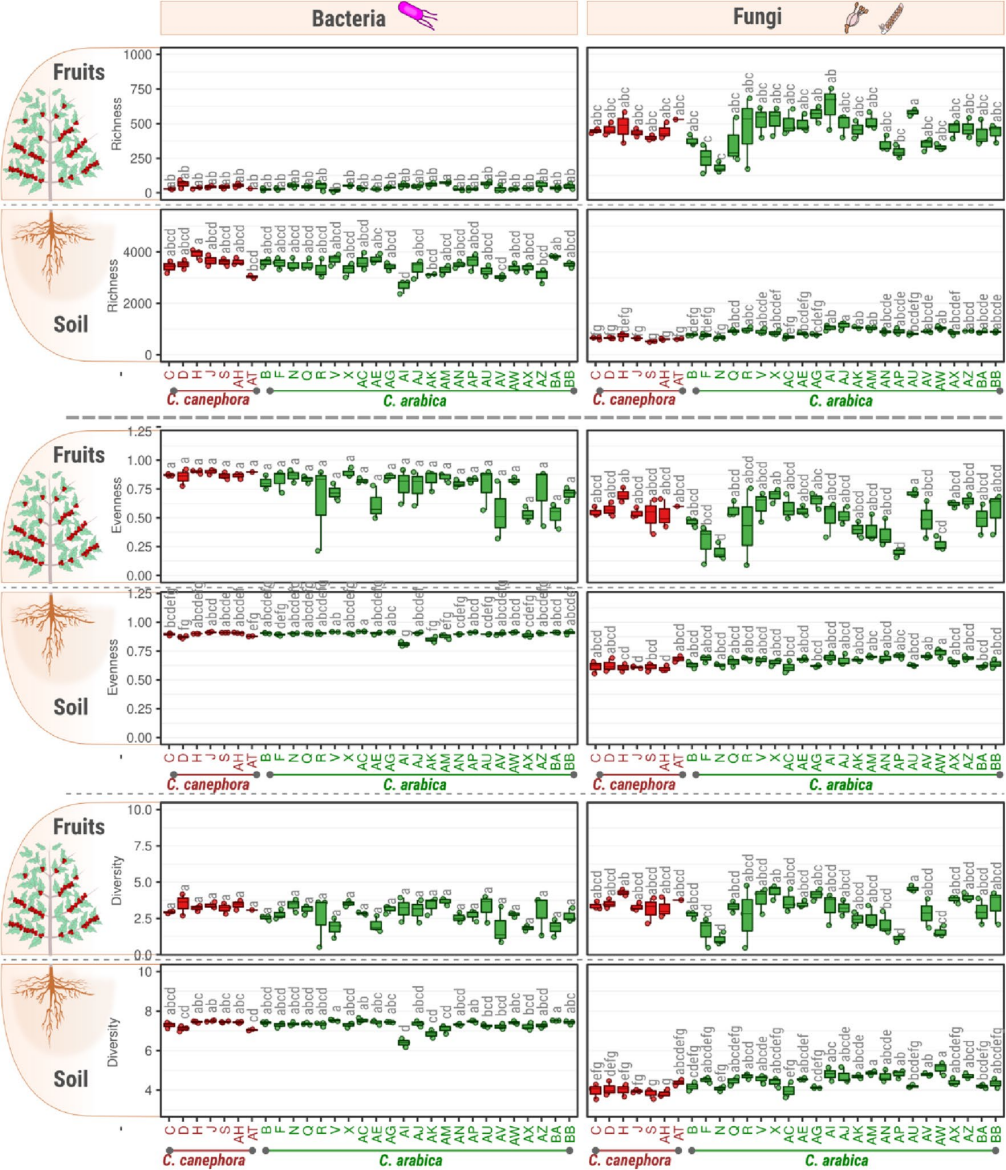
Alpha-metrics of bacterial (left plot) and fungal (right plot) communities harbored in fruits and soils of coffee plants from two species: *Coffea arabica* (green) and *Coffea canephora* (red). The letters above each boxplot were calculated using the Kruskal–Wallis’ test. Areas with the same letter do not have statistical differences.

Regardless of the alpha metrics between species, the richness, evenness, and diversity of soil bacteria were higher than the fungal measurements, while in fruits the bacterial richness was lower than that of fungi (Fig. 5A). This same pattern was also observed across the floristic domains (Fig. 5B). However, it is worth noting that most of the sequencing effort in fruits was lost by the amplification of sequences from mitochondria and chloroplasts, therefore the observations of the bacteria in fruits should be done with caution. We did not find significant differences in the alpha diversity metrics among species. As expected, the highest values of diversity were observed in the coffee plantation under organic management (property AW, Fig. 6) and the lowest values of bacterial diversity were found in plantations where extractivism without management is performed (property AI). We also found that the fungal diversity in soils and fruits were inversely correlated (r_pearson_ = − 0.37; p-value = 0.045).

### Taxonomic composition

**Figure 7:**
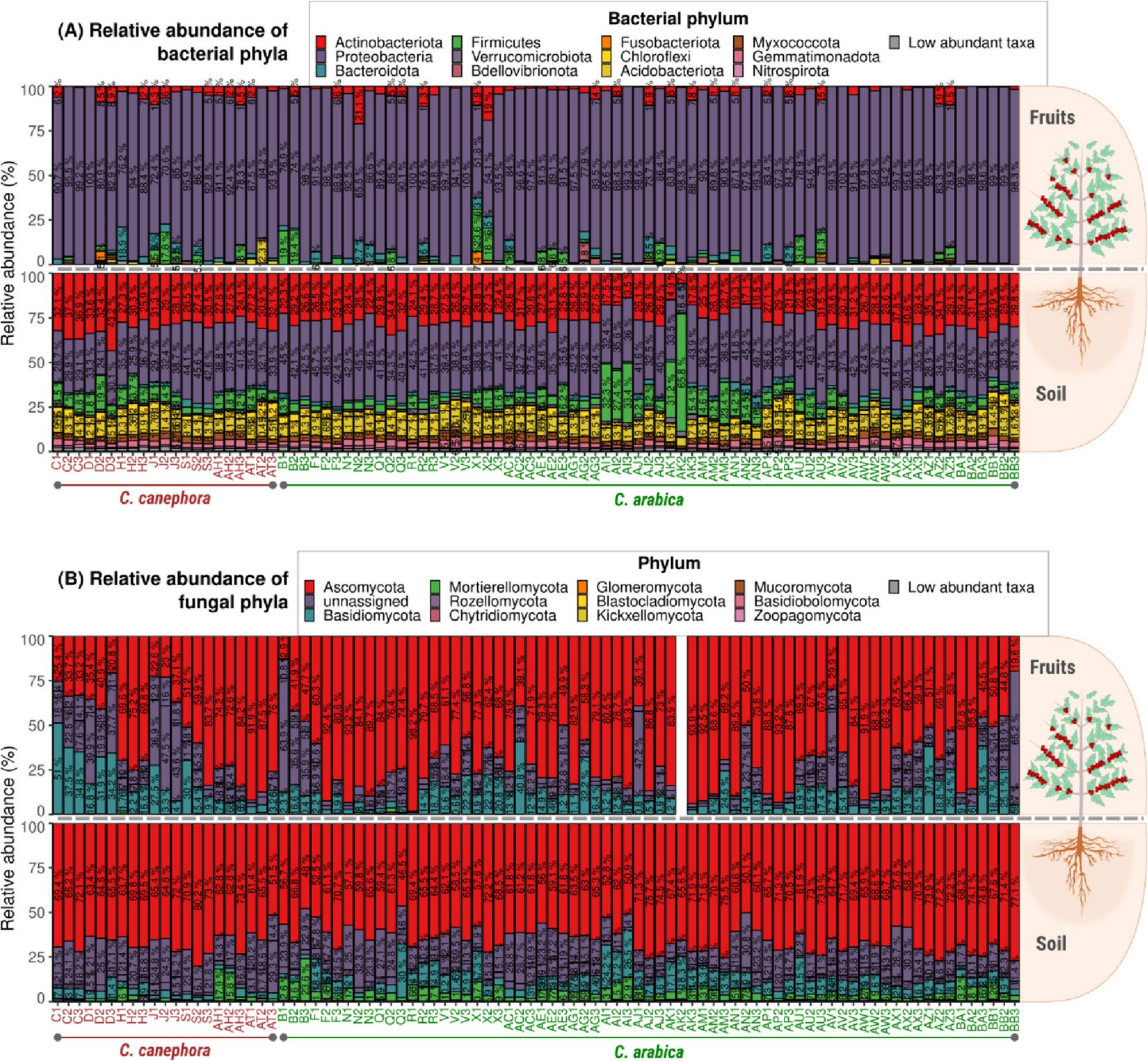
Taxonomic composition at the phylum level in soils of each coffee plantation. Values within bars represent the relative abundance of each phylum based on the 16S and ITS1 sequencing.

We detected 14 bacterial phyla with abundance equal to or above 1% in the fruit samples (Fig. 7A). The most abundant were Proteobacteria (more than 50%), Actinobacteria (1.3–21.4%), Firmicutes (1.3–25.2%), and Bacteroidota (1.3–18.4%). The abundance of other phyla such as, Verrucomicrobiota, Fusobacteriota, Myxococcota, Armatimonadota, Chloroflexi, Bdellovibrionota, Acidobacteriota, Campilobacterota, Phragmoplastophyta, and Deinococcota, was in the lower in fruits. The most abundant fungal phyla were Ascomycota (23.3–98.5%), Basidiomycota (1–62.5%), and Mortierellomycta (1.9–2.1%). Rozellomycota, Chytridiomycota, Glomeromycota, Monoblepharomycota, Blastocladiomycota, Mucoromycota, Basidiobolomycota, Olpidiomycota, Kickxellomycota, Neocallimastigomycota, and Entorrhizomycota accounted for less than one percent (Fig. 7B).

In the soil samples, 41 bacterial phyla were detected. The ten most abundant were Proteobacteria (Fig. 7A), which composed more than 60% of sequences identified (63.6% ± 5.84), Actinobacteria (27.4% ± 5.57), Acidobacteriota (9.1% ± 1.99), Firmicutes (8.7% ± 8.92), Chloroflexi (4.1% ± 1.69), Myxococcota (3.3% ± 0.80), Gemmatimonadota (3.0% ± 1.03), Bacteroidota (2.1% ± 1.0), Verrucomicrobiota (1.3% ± 0.55), and Nitrospirota (0.9% ± 0.26). However, the most abundant fungal phyla were Ascomycota (63.6% ± 7.49), Basidiomycota (7.1% ± 5.32), and Mortierellomycta (4.9% ± 4.00). Other phyla such as Rozellomycota, Chytridiomycota, Glomeromycota, Basidiobolomycota, Mucoromycota, and Zoopagomycota accounted for less than one percent of the reads (Fig. 7B).

Some specific taxa have shown a distinct relative abundance related to the floristic domain or to the coffee species. For instance, the genus *Saitozyma* displayed higher relative abundances in soils of *C. arabica* (mean relative abundance = 4.13% ± 3.56) than in soils of *C. canephora* (0.17% ± 0.23) (Fig. S2). Furthermore, *Bacillus bataviensis* had the highest relative abundance in the soils of the Caatinga floristic domain (Properties AI, AK, and AM; Fig. S3). The most abundant nitrogen-fixing bacterial group found in the soils of *C. arabica* was the *Bradyrhizobium* genus (Proteobacteria Phyla, Fig. S3) while in *C. canephora*, *Sphingomonas* was the main genus found. As shown before (Fig. 3A), fungal composition differed greatly between the coffee species. The main fungi found in both species could not be identified to deeper levels of classification (i.e. species, genus, and families).

We detected 356 Amplicon Sequence Variants (ASVs) from the phylum Glomeromycota that are Arbuscular Mycorhiza Fungi (AMFs) (Fig. S4). These were clustered into 191 operational taxonomic units (OTUs). Most OTUs could not be identified to the genus or species level, with most of them being identified only to the order or family level. Furthermore, only ten OTUs of AMFs were shared between both *Coffea* species, and the remaining were associated with only one coffee species. Some of these OTUS were observed exclusively in locations with water shortages.

Yeasts were also evaluated in coffee fruits (Fig. S5). *Candida* sp.*, Pichia* sp.*, Rhodotorula* sp.*, Saccharomycopsis* sp.*, Sporopachydermia* sp.*, Debaromyces* sp.*, Kluveromyces* sp.*,* and *Hanseniaspora* sp. were observed in both species produced in the different floristic domains (Fig. S5A)*.* The genus *Blastobotrys* was found exclusively in *C. canephora* and seems to be exclusive of the Atlantic Forest (Fig. S5B).

## Discussion

This study is the first to report microbial communities associated with Brazilian coffee produced in four floristic domains (Amazon, Atlantic Forest, Caatinga, and Cerrado) and eight states (PR, SP, MG, ES, BA, CE, PE, and RO). The amount of data generated by DNA sequencing was enough to assess the microbial diversity in fruits and soils of coffee plants (Supplementary Table 1), while the large amount of data provides new insights into microbial roles and their potential for the development of more sustainable coffee management. For instance, the production of microbial inoculants that help plant nutrition (biofertilizers), reduction of the use of agrochemicals, and in fermentation of coffee cherries.

The distinct microbial community associated with each coffee species and floristic domain observed in this study (Fig. 3) may be due to the rhizosphere effect of the host. However, we cannot consistently assert that the differences found in both communities were exclusively due to the *Coffea* species because this factor is mixed up with the environmental conditions under which each species grows. For instance, the cultivation altitudes of the two coffee species are different. *C. arabica* is cultivated at high altitudes, while *C*. *canephora* plantations are usually located at low altitudes. The samples of this study were obtained at altitudes below 553 m for *C. canephora* and above 763 m for *C. arabica*. It is interesting to note that the microbial communities associated with the rhizosphere of *C. canephora* at the highest elevation (553 m) were more similar to the microbial communities associated with the rhizosphere of *C. arabica* at the lowest elevation (763 m). Furthermore, these two crops are located close to each other (approximately 18 km) and other studies have shown that altitude is one of the main factors driving the microbial composition of coffee soils^6,11^. Moreover, other factors like the management system^12^, the phenological stages of coffee^12,13^ and the chemical composition of soil can also modulate the edaphic microbial community^6^.

The high relative abundances of *Bacillus bataviensis* in the soils from the Caatinga (Properties AI, AK, and AM) may be due to the characteristics of adaptations and survival of this microorganism in soils with low water availability. The increased relative abundance of this genus was related to increased plant resilience during drought stress^14–16^. The Caatinga region has low pluviometric indices and the presence of soil microorganisms that improve plant resilience may be one of the keys that enables the growth of coffee plants under these harsh conditions. Furthermore, the relatively high abundance of *Saitozyma* in soils of *C. arabica* may be related to the content of organic matter. This fungus has the potential to degrade plant-derived lignocellulosic compounds in the soil and carbohydrates with five carbon atoms^17^. According to Aliyu et al.^17^, in the *Saitozyma* sp. growth on D-xylose there is an overrepresentation of genes for the D-xylulose reductase/L-iditol 2-dehydrogenase. The degradation of xylose produces xylitol, which is used as raw material in the sweetener production with a low glycemic index. Thus, the *Saitozyma* sp. has potential to be used as starter culture in coffee fermentation to increase the sensory perception of sweetness in the coffee beverage.

One of the most abundant bacterial ASVs able to fix nitrogen observed in both *Coffea* species was *Bradyrhizobium* sp. (Fig. S3). This result corroborates recent studies of the microbiome associated with the soils of coffee plants^18,19^. Furthermore, other genera, for example *Sphigomonas* and *Sphingobium* (Fig. S3) related to nitrogen fixation are present in soils of *C. arabica*^20^. However, there are still no commercial inoculants of nitrogen-fixing bacteria for coffee planting, which shows the potential for bioprospecting of the native coffee microbiota.

A high number of ASVs (191 OTUs at 97% threshold) were identified as belonging to the Glomeromycota (Fig. S4). However, it is important to note that the relative abundance of these fungi was below one percent in samples. Although ITS1F/ITS4 are often a suitable pair of primers to estimate the overall fungal diversity of samples, some authors argue that this primer set used in DNA amplification has a great impact on the diversity of AMF detected by the Next Generation Sequencing^21,22^. The AMFs have an important role in plant growth and yield because they can improve soil fertility and water and nutrient uptake by plants^2^, which is very important in coffee production under conditions of water stress like those observed in Caatinga soil. Furthermore, the presence of these fungi has been shown to benefit from agroecological management^12^ which is mostly due to the reduction in the amount of the chemical fertilizers used.

The area with organic management (AW) had the highest fungal diversity (Fig. 6) and the most distinct bacterial beta diversity (Fig. 4) among the 30 plantations evaluated in this study. Variations in microbial diversity were greater for the fungal community than for other microbial communities under different types of management^12,23^. The use of conventional agrochemical management may reduce microbial diversity due to the toxic effects of these products^24^. However, the lowest bacterial diversity was observed in coffee planted within forests under extractivism without management (site AI, Fig. 6). The lack of management in extractivism may compromise the condition of the soil, leading to negative impacts on bacteria. Therefore, crop management has a high impact on soil microbial diversity.

Because of the distinct fungal communities harbored by fruits of each coffee species (Fig. S3), we investigate if these species could harbor yeasts with potential for use as starter culture in the fermentation of coffee fruits. Coffee fermentation has been used to improve the chemical and sensory quality of *C. arabica* and *C. canephora* fruits^4,25,26^. In this technique, a starter culture (e.g. *Saccharomyces cerevisiae*) is added into the fermentation tank containing fruits or beans of coffee^4,25^. Although most of ASV yeasts have not been identified to the species level, some of them are found exclusively in one of the two coffee species or in only one of the floristic domains (Fig. S5). This result highlights the importance of testing wild yeasts of coffee cherries in fermentation as these microorganisms may be more adapted to the natural condition of the coffee-growing region and provide better flavors to the coffee beverage. The use of wild yeasts in fermentation of fruits other than coffee have shown potential for producing beverages with more desirable sensory attributes than those of the commercial strains^26^.

This broad survey performed across the Brazilian coffee producing regions showed that the genus *Coffea* harbors a high diversity of microbial communities associated with fruits and soil. There is a clear difference between the microbial communities associated with *C. arabica* and *C. canephora* in each floristic domain. Furthermore, within each species there is also variation among the microbial communities in the planting region. Many fungi and bacteria detected that have not been cultivated yet show potential for application in pre- and post-harvesting of coffee production (Figs. S4 and S5). Nitrogen-fixing bacteria and AMFs may be used in pre-harvesting to increase plant growth and fruit production, while the yeasts and bacteria had potential to be applied in coffee fermentation. Therefore, this opens up an opportunity to better explore the microbial potential for the development of a sustainable coffee production chain.

## Methods

### Study area

A total of 180 samples (90 from soil and 90 from fruits) were collected from 30 plantations (seven of *C. canephora* and 23 of *C. arabica*; Table 1) of eight Brazilian states located in four floristic domains (Amazon, Atlantic Forest, Cerrado, and Caatinga; Fig. 1). The sampling process was performed as described by Veloso et al., 2020. The greater number of samples of *C. arabica* than of *C. canephora* is due to the greater production of *C. arabica* across the country and in the study sites.

**Table 1:** Coffee farms in Brazil (30 farms) used in this study to evaluate the coffee microbiome.

Site ID	Altitude (m)	Coffee species	Brazilian s tate	Municipality	Management type	Genotype
C	167	*C. canephora*	ES	São Gabriel da Palha	Conventional	Vitória
D	231	*C. canephora*	ES	São Domingos do Norte	Conventional	Vitória
H	200	*C. canephora*	ES	Jerônimo Monteiro	Conventional	Verdim
J	427	*C. canephora*	ES	Conceição do Castelo	Conventional	Vitória
S	185	*C. canephora*	BA	Teixeira de Freitas	Conventional	Vitória
AH	572	*C. canephora*	ES	Afonso Cláudio	Conventional	Unknown
AT	249	*C. canephora*	RO	Cacoal	–	Unknown
B	719	*C. arabica*	ES	Afonso Claúdio	Conventional	Catuai 81
F	788	*C. arabica*	ES	Venda Nova do Imigrante	Conventional	Catuaí vermelho—44
N	1113	*C. arabica*	MG	Serra do Salitre	Conventional	Catuaí Vermelho
Q	1087	*C. arabica*	MG	Campanha	Conventional	Mundo Novo
R	1021	*C. arabica*	MG	Campanha	Agroforestry	Catuaí Amarelo—2SL
V	743	*C. arabica*	RJ	Porciuncula	Conventional	Japi e Asa Branca
X	1037	*C. arabica*	ES	Afonso Claúdio	Conventional	Catucaí 785
AC	973	*C. arabica*	MG	Três Pontas	Conventional	Arara Amarelo
AE	917	*C. arabica*	SP	Pedregulho	Conventional	Catuaí Amarelo—2SL
AG	832	*C. arabica*	ES	Domingos martins	Conventional	Catuai Amarelo
AI	672	*C. arabica*	CE	Pacoti	in forest with extractivism without management	Desconhecido/Typica
AJ	813	*C. arabica*	CE	Mulungu	Agroforestry	Acauã Novo
AK	874	*C. arabica*	CE	Guaramiranga	Agroforestry	Desconhecido/Mundo Novo
AM	897	*C. arabica*	PE	Exu	Conventional	Catuaí
AN	1184	*C. arabica*	BA	Piatã	Conventional	Catuaí—144
AP	984	*C. arabica*	MG	Espera Feliz	Conventional	Catiguá
AU	922	*C. arabica*	BA	Vitória da Conquista	Conventional	Unknown
AV	1264	*C. arabica*	SP	Caconde	Conventional	Sumatra/Typica
AW	1236	*C. arabica*	MG	Araponga	Organic	Catuaí Vermelho—44
AX	771	*C. arabica*	PR	Congoinhas	Coventional	IPR100
AZ	911	*C. arabica*	MG	José Gonçalves de Minas	Coventional	Catuaí Vermelho
BA	1032	*C. arabica*	ES	Ibitirama	Coventional	Unknown
BB	1008	*C. arabica*	ES	Castelo	Conventional	Catuaí 81

A total of three samples per farm was used for all the analyses.

### DNA extraction and PCR

Aliquots of 250 mg of each sample were added to NucleoSpin^®^ Bead Tubes Type A (containing ceramic beads) with 700 μL of SL1 buffer and 150 μL of Enhancer SX provided by the kit Nucleospin Soil^®^ (Machanarey-Nagel)^27^. Cell lysis was carried out in a Precellys 24 High-Powered Bead Mill Homogenizer (Bertin technologies) for 50 s at 4000 rpm. DNA extraction was performed according to the manufacturer’s protocol. The quality of DNA extraction was evaluated by electrophoresis in 0.8% agarose gel stained with ethidium bromide under UV light.

The region V3-V4 of *16S rDNA* gene was amplified with the primers 341F (5’-CCTACGGGAGGCAGCAG-3’) and 806R (5’-GGACTACNVGGGTWTCTAAT-3’)^28^. The Internal Transcribed Spacer 1 (ITS1) fungi were amplified with the primers ITS1F (5’-CTTGGTCATTTAGAGGAAGTAA-3’) and ITS2 (5’-GCTGCGTTCTTCATCGATGC-3’)^29^. The PCR reactions were performed with Phusion^®^ High-Fidelity PCR Master Mix (New England Biolabs) and specific barcodes were used for each sample. The PCRs were evaluated in a 2% agarose gel using SYBR green. Samples with the main bright strip between 400 and 450 bp were used for downstream experiments. The PCR were mixed in equally dense ratios and purified with Qiagen Gel Extraction Kit (Qiagen, Germany).

The sequencing libraries were prepared using NEBNext^®^Ultra™ DNA Library Prep Kit for Illumina according to the manufacturer's recommendations. The library quality was evaluated on a Qubit@ 2.0 Fluorometer (Thermo Scientific) and Agilent Bioanalyzer 2100 system. Finally, the Illumina NovaSeq 6000 platform was used to sequence the libraries to produce 250 bp paired-end reads.

### Bioinformatic analyses

The raw reads were demultiplexed and trimmed to remove primers and adapters. All those with a maximum number of expected error (*maxee*) equal or greater than one and the chimeras or singletons were removed. The remaining sequences were used to determine the Amplicon Sequence Variants (ASVs) using the Divisive Amplicon Denoising Algorithm (DADA2)^30^⁠. The SILVA 138 database was used to annotate the ASVs. The ASVs of the DNA from cell organelles (mitochondria and chloroplasts) were removed from the downstream analyses. All the steps described above were performed using QIIME2 2021.4^31^.

The beta-diversity of the total microbial community was evaluated by Principal Coordinate Analysis (PCoA) based on the Bray Curtis’ distance between samples using the read abundances of 16S rDNA and ITS. The alpha diversity metrics (Richness, Pielou’s evenness, and Shannon’s diversity) were calculated using the package microbiome^32^.

### Complies with international, national and/or Institutional guidelines

The experimental research on plants of this study complies with relevant institutional, national, and international guidelines and legislation.

### Permissions to collect the samples

We have permission to collect all the samples in this survey. This study was registered in a Brazilian Platform (Plataforma Brasil: https://plataformabrasil.saude.gov.br/login.jsf) with the number CAAE 31567320.2.0000.5072.

### Identification of plants

All the plants were identified by specialists from the *Instituto Capixaba de Pesquisa, Assistência Técnica e Extensão Rural* (Incaper) that have expertise in coffee cultivation. No voucher material of these specimens was deposited because it does not apply to them as they are plant of commercial usage.

### Supplementary Information

Supplementary Information.

## Data Availability

All the raw sequences were deposited in the Sequence Read Archive (SRA) of the National Center for Biotechnology Information (NCBI) and can be accessed using the accession number PRJNA626678.
