# Supplementary material for: Microbiomes associated with Coffea arabica and Coffea canephora in four different floristic domains of Brazil
Source: Sci Rep. 2023 Oct 28;13:18477. doi: 10.1038/s41598-023-45465-w (PMC10613301; doi:10.1038/s41598-023-45465-w)
Supplement: Supplementary file 1 — Supplementary Information. [file 41598_2023_45465_MOESM1_ESM.pdf]

Supplementary information

Microbiomes associated with *Coffea arabica* and *Coffea canephora* in four floristic domains of Brazil

Tomás Gomes Reis Veloso[2], Marliane de Cássia Soares da Silva[2], Tais Rizzo Moreira[3], José Maria Rodrigues da Luz[2], Aldemar Polonini Moreli[1], Maria Catarina Megumi Kasuya[2], Lucas Louzada Pereira[1*]

[1]Instituto Federal do Espírito Santo. Coffee design. Avenida Elizabeth Minete Perim, S/N, Bairro São Rafael, Venda Nova do Imigrante, CEP: 29375-000, Espírito Santo, Brazil.

[2]Universidade Federal de Viçosa, Departamento de Microbiologia, Laboratory of Mycorrhizal Associations – LAMIC. Avenida Ph Rolfs S/N, Viçosa, CEP:36570-000, Minas Gerais, Brazil.

[3]Universidade Federal do Espírito Santo. Centro de ciências agrárias e engenharias. Av. Gov. Lindemberg, 316 - Centro, Jerônimo Monteiro, CEP: 29550-000, Espírito Santo, Brazil.

*** Correspondence:**
Correspondence Corresponding author: Lucas L. Pereira. Federal Institute of Espirito Santo, Avenida Elizabeth Minete Perim, Venda Nova do Imigrante, ES, Brazil. Zip Code 29375-000. Orcid: 0000-0002-4436-8953. E-mail address: lucaslozada@hotmail.com

Table S1: Sampling effort based on the coverage index (Chao & Jost, 2012).

Property ID	Fruit		Soil	
	**16S**	**ITS1**	**16S**	**ITS1**
**AC**	1.0000	1.0000	0.9984	1.0000
**AE**	0.9999	1.0000	0.9987	1.0000
**AG**	0.9997	1.0000	0.9983	1.0000
**AH**	0.9997	1.0000	0.9985	1.0000
**AI**	0.9994	1.0000	0.9990	1.0000
**AJ**	0.9995	1.0000	0.9986	1.0000
**AK**	0.9996	1.0000	0.9989	1.0000
**AM**	0.9995	1.0000	0.9989	1.0000
**AN**	0.9999	1.0000	0.9987	1.0000
**AP**	1.0000	1.0000	0.9988	1.0000
**AT**	1.0000	1.0000	0.9990	1.0000
**AU**	0.9996	1.0000	0.9989	1.0000
**AV**	0.9999	1.0000	0.9987	1.0000
**AW**	1.0000	1.0000	0.9988	1.0000
**AX**	0.9998	1.0000	0.9986	1.0000
**AZ**	0.9998	1.0000	0.9986	1.0000
**B**	0.9998	1.0000	0.9986	1.0000
**BA**	0.9999	1.0000	0.9985	1.0000
**BB**	0.9997	1.0000	0.9985	1.0000
**C**	1.0000	1.0000	0.9985	1.0000
**D**	0.9997	1.0000	0.9986	1.0000
**F**	1.0000	1.0000	0.9984	1.0000
**H**	0.9997	1.0000	0.9982	1.0000
**J**	0.9991	1.0000	0.9983	1.0000
**N**	0.9999	1.0000	0.9986	1.0000
**Q**	0.9998	1.0000	0.9985	1.0000
**R**	1.0000	1.0000	0.9986	1.0000
**S**	1.0000	1.0000	0.9985	1.0000
**V**	1.0000	1.0000	0.9983	1.0000
**X**	0.9994	1.0000	0.9986	1.0000

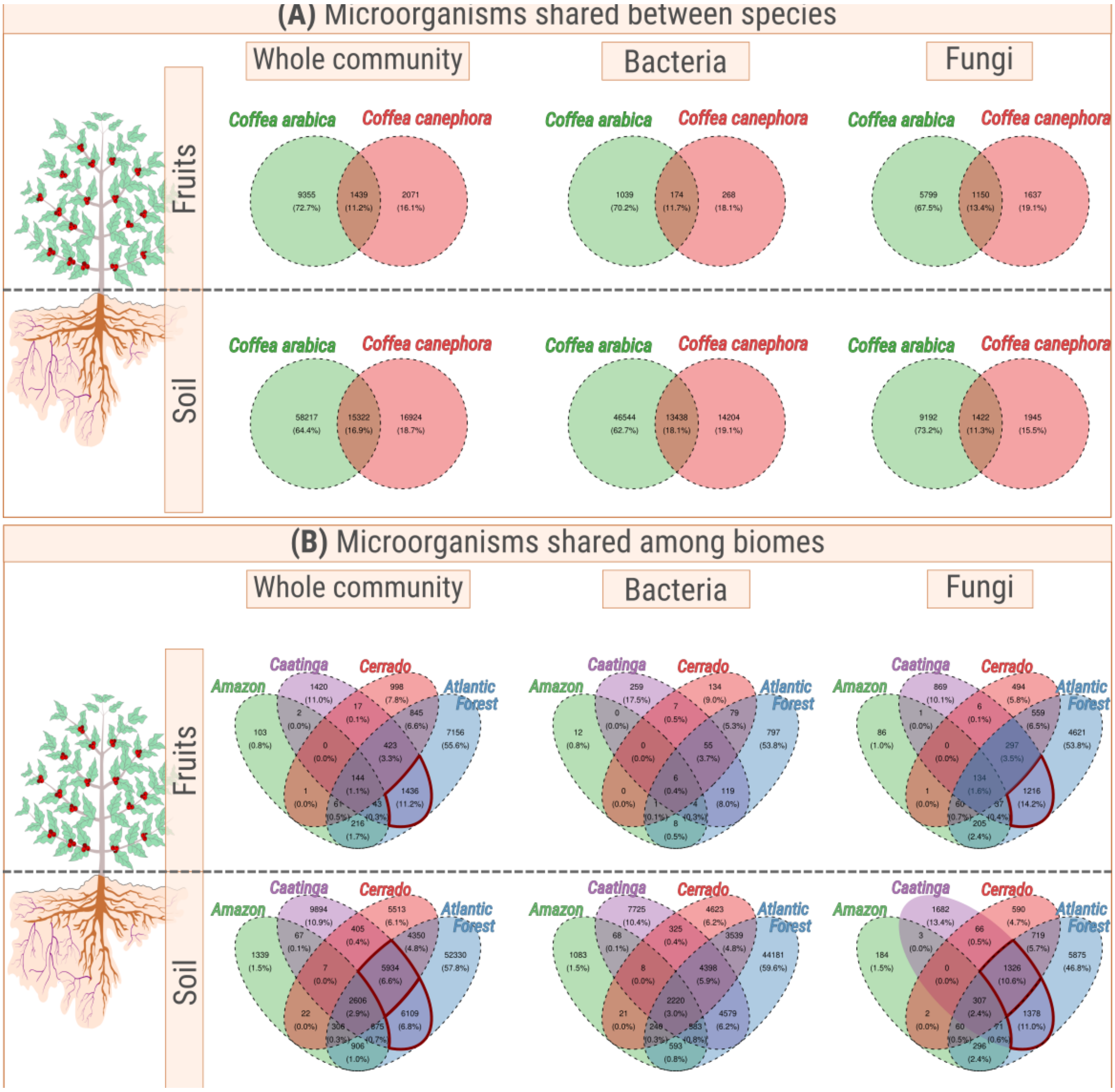

Supplementary figure S1: Number of shared ASVs across the (A) *Coffea* species (*Coffea arabica* and *Coffea canephora*) and (B) floristic domains (Atlantic Forest, Caatinga, Cerrado and Amazon).

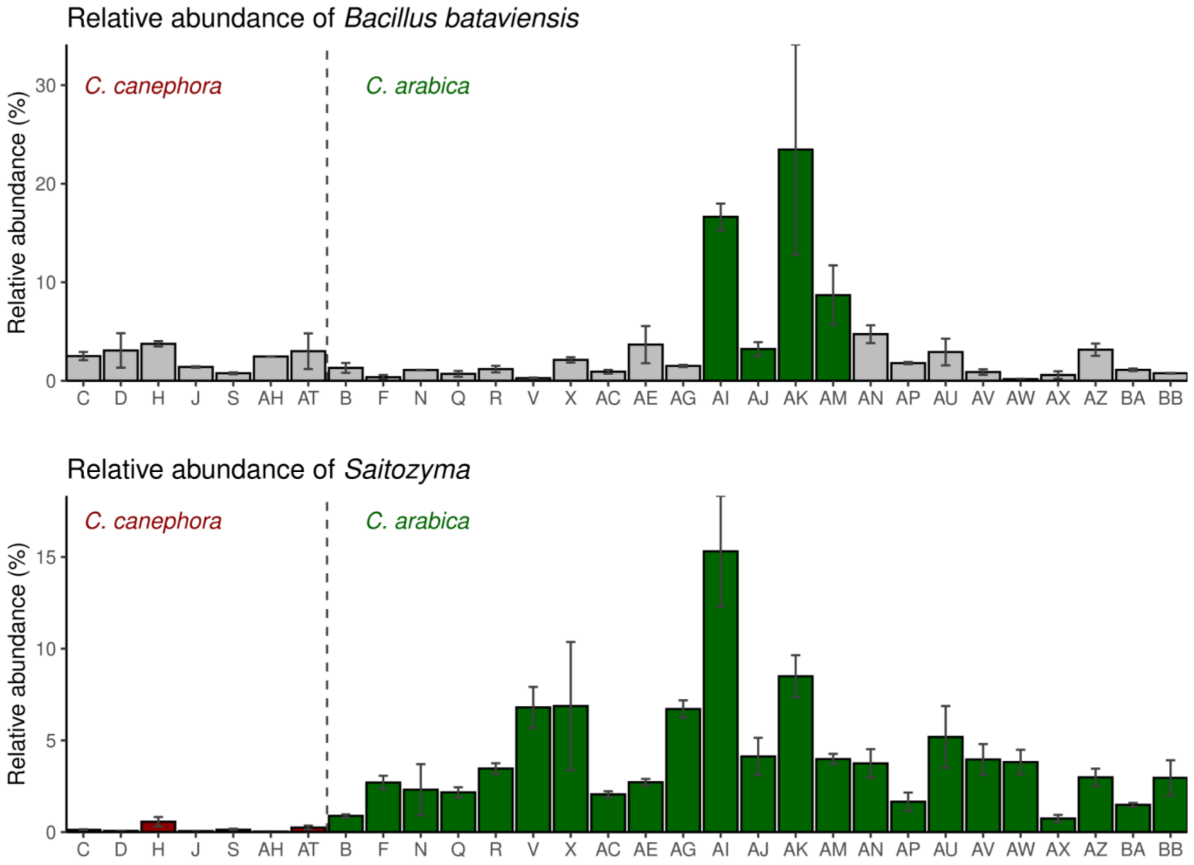

Supplementary figure 2: Relative abundances of *Bacillus Bataviensis* and *Saitozyma* sp. in soil samples from crops of *Coffea arabica* and *Coffea canephora* crops of *Coffea* in the Brazilian floristic domains (Amazon, Caatinga, Cerrado, and Atlantic Forest).

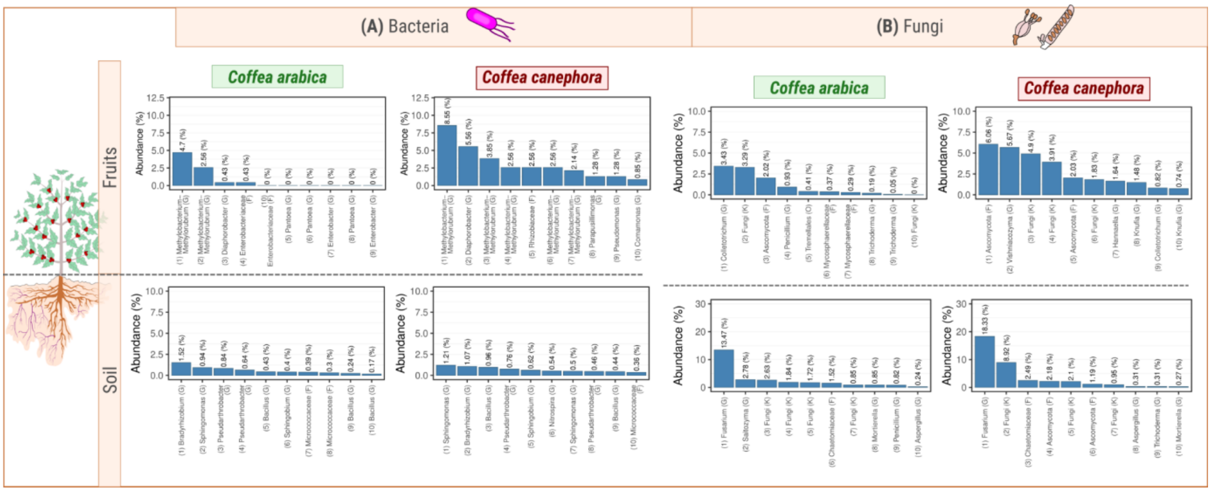

Supplementary figure 3: Relative abundances of microorganism in soil and fruit samples from crops of *Coffea arabica* and *Coffea canephora* in the Brazilian.

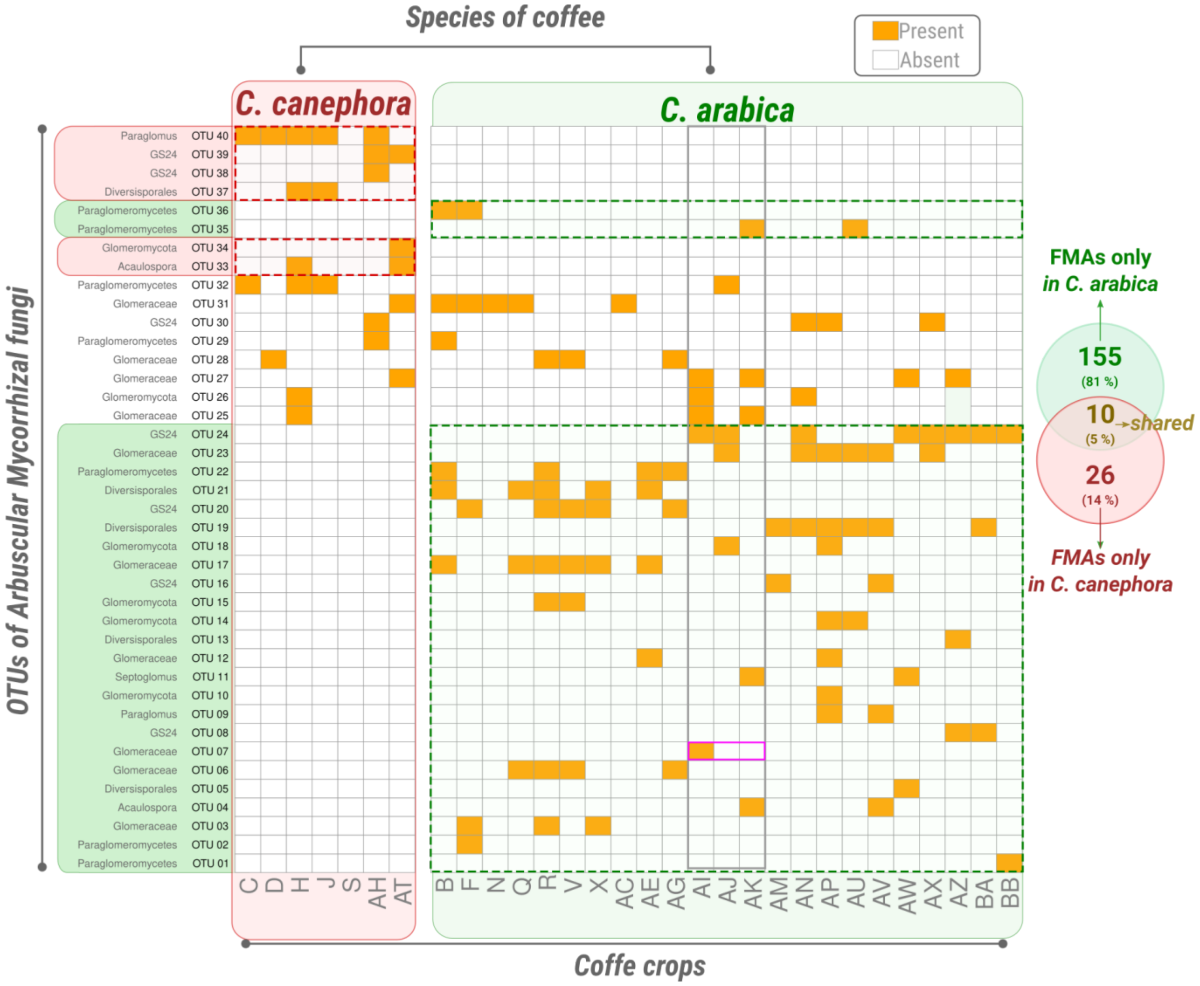

Supplementary figure 4: The 40 most abundant Operational Taxonomic Units (OTUs) of Arbuscular Mycorrhizal fungi (AMF) found in the soil of *Coffea canephora* and *Coffea arabica*. OTUs' names highlighted with green or red represent OTUs found exclusively in one coffee species. OTUs highlighted with green or red were found exclusively in one coffee species. The Venn diagram shows the percent of unique and shared OTUs of FMAs between the two species.

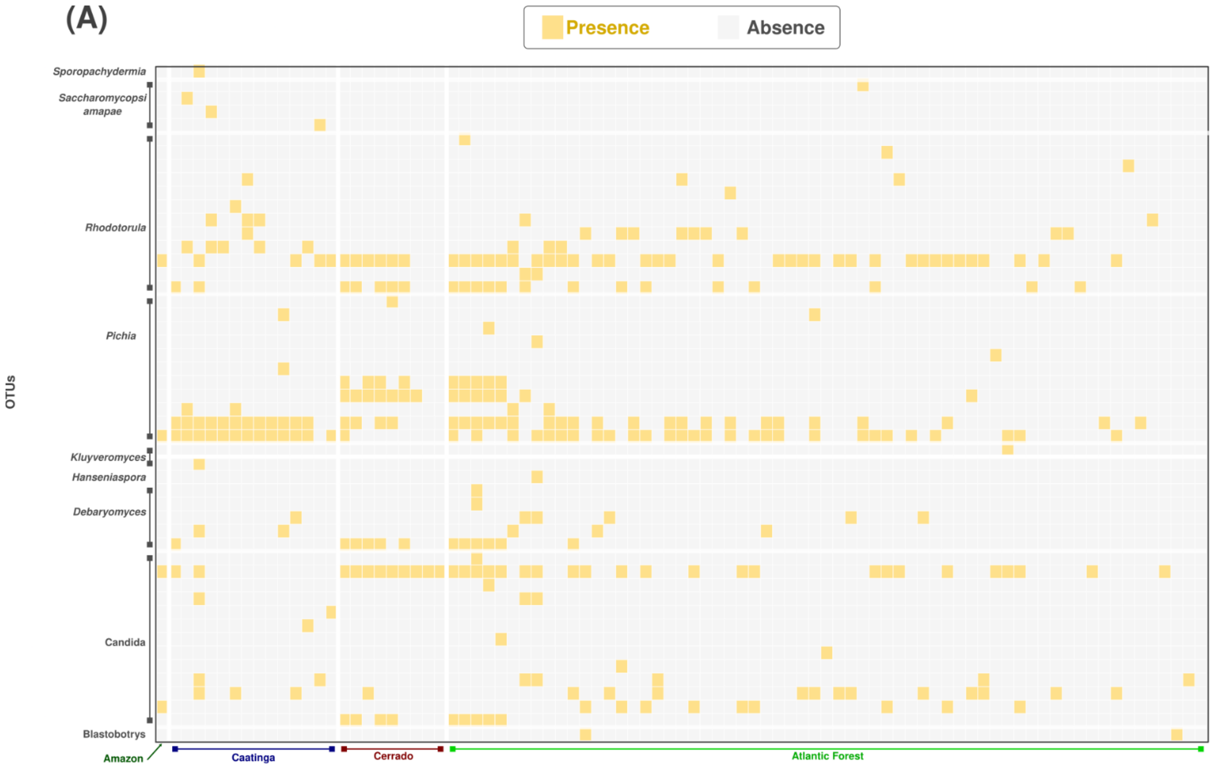

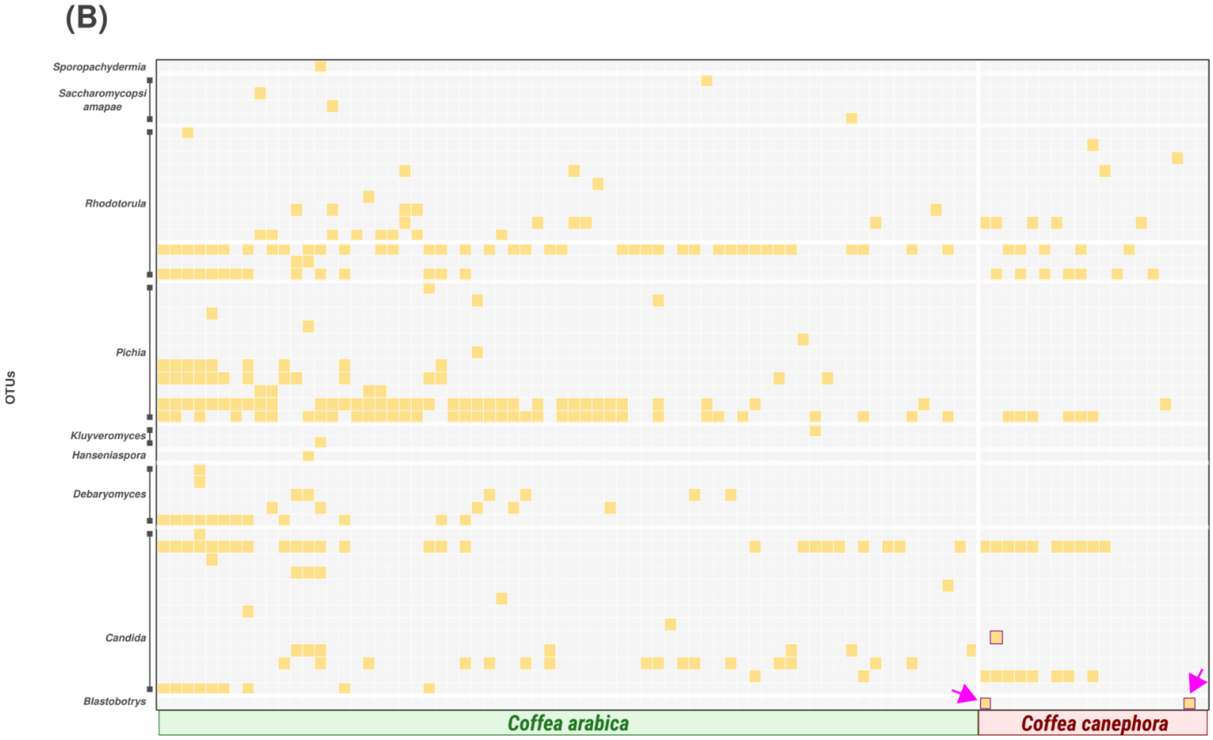

Supplementary figure 5:Operational Taxonomic Units (OTU) of yeasts found in fruits of *C. arabica* and *C. canephora* in (A) different floristic domains and (B) *Coffea* species. OTUs of *Blastobotrys* (pink arrows) were found only in samples of *Coffea canephora*.
